# Continuation with clozapine after eosinophilia: a case report

**DOI:** 10.1186/s12991-017-0169-8

**Published:** 2017-12-14

**Authors:** Yen-Cheng Ho, Huang-Li Lin

**Affiliations:** 1 Department of Psychiatry, Chang-Gung Memorial Hospital at Linkuo, Taoyuan City, Taiwan; 2 Chang-Gung University School of Medicine, Taoyuan City, Taiwan

**Keywords:** Clozapine, Eosinophilia, Schizophrenia

## Abstract

**Background:**

Clozapine-induced eosinophilia had been reported in several studies about blood dyscrasias in clozapine-treated patient. The largest study with 2404 patients in Italy found the incidence of 2.2% by criteria of more than 0.4 × 10^9^/l. Associated cases of pancreatitis, hepatitis, colitis, nephritis, and myocarditis were reported. Interestingly, incidence of myocarditis is high in Australia, but low in the rest of the world. In the following, we will present a case of clozapine-induced eosinophilia which spontaneous resolution was noted under continuation of clozapine.

**Case presentation:**

“Mr. L” was a 54-year-old single, jobless man. He had treatment-resistant chronic schizophrenia with onset at age 28. He had received electroconvulsive therapy twice prior to this admission. After admission, a trial of clozapine was started with an initial dose of 100 mg/day, and gradually titrated to 200 mg/day. He experienced notable improvement after 2 weeks with decreased auditory hallucinations and no more self-harm behaviors, but he also developed eosinophilia. A medical workup was performed and showed no signs of end-organ inflammation. We continued clozapine use and closely monitored complete blood count with a differential test to track his eosinophil count by the recommendation of the hematology service. His eosinophil count decreased then and remained within normal limits 3 weeks later. The dosage of clozapine was gradually raised as high as 400 mg/day. His psychotic symptoms got partial remission and continued to show no signs of end-organ inflammation at the time of discharge.

**Conclusions:**

The pathophysiology of clozapine-induced eosinophilia is still unknown, but resolution of eosinophilia despite ongoing clozapine treatment suggests the possibility of an acute allergic reaction. Signs or symptoms of organ inflammation are important for management of eosinophilia. In this case report, we demonstrated that if eosinophilia occurred without signs or symptoms of organ inflammation, it may be justified to continue clozapine use under careful monitoring.

## Background

Clozapine-induced eosinophilia had been reported in several studies about blood dyscrasias in clozapine-treated patient [1–6]. The largest study with 2404 patients in Italy found the incidence of 2.2% by criteria of more than 0.4 × 10^9^/l [1]. Associated cases of pancreatitis [7, 8], hepatitis [9, 10], colitis [11, 12], nephritis [13, 14], and myocarditis [15] were reported. Interestingly, incidence of myocarditis is high in Australia, but low in the rest of the world [15]. In the following, we will present a case of clozapine-induced eosinophilia which spontaneous resolution was noted under continuation of clozapine.

## Case presentation

**“**Mr. L” was a 54-year-old single, jobless man. He had treatment-resistant chronic schizophrenia with eight previous psychiatric hospitalizations, mostly in recent 2 years prior to this admission in 2012, and had been received electroconvulsive therapy (ECT) twice. He also had type 2 diabetes mellitus under medical control. There were no other systemic diseases.

After completing military service, Mr. L worked as a carpenter for several years. At age 28, unstable mood and violent behavior were first noted by his family. He was hospitalized and diagnosed with schizophrenia, presented with delusion of persecution, misidentification, delusion of reference, and auditory hallucination. Under medications, he could keep daily function and housework, but he never returned to his baseline functionality.

One year prior to this admission, Mr. L could not keep housework and self-care due to aggravated delusion of persecution, reference, and auditory hallucination. He had a suicide attempt by trying to explode a gas tank, and then he became one of the recurrently readmitted inpatient psychiatric patients. He had poor response to high dosages of risperidone, zotepine, quetiapine, olanzapine, paliperidone, haloperidol, and even flupenthixol with another oral antipsychotic medication combination trial. He was ever prescribed clozapine in other hospitals for short period of time, but discontinued without blood dyscrasias. After two times of ECT, he showed more stationary condition so he was transferred to our psychiatric chronic ward. However, medication refusal and aggravated hallucinatory behavior were noted 2 months after last ECT was done. He would shout, follow a specific female worker, and have self-harm behaviors such as slapping and kowtowing due to commanding auditory hallucination.

After admission, a trial of clozapine was started with initial dose of 100 mg/day, and gradually titrated to 200 mg/day, with concomitant treatment of flupenthixol 40 mg intramuscular injection every 2 weeks, benzodiazepines, laxatives, and one oral hypoglycemic agent. Moderate drooling was observed but did not influence his intake. He experienced notable improvement after 2 weeks with decreased auditory hallucinations and no more self-harm behaviors, but he also developed eosinophilia, with a count of 1.4 × 10^9^/l, 15.5% of the total WBC count (9.3 × 10^9^/l). Eosinophilia persisted 4 days later, with a count of 3.8 × 10^9^/l, 32% of the total WBC count (12.0 × 10^9^/l).

Before deciding whether to continue the clozapine treatment, we reviewed the published literature about eosinophilia after clozapine use [16], and successful rechallenge had been reported. A medical workup was performed, including cardiac enzymes, an electrocardiography, liver function tests, blood creatine level, blood urea nitrogen, levels of amylase, lipase, erythrocyte sedimentation rate, and C-reactive protein, none of which showed evidence of myocarditis, hepatitis, nephritis, pancreatitis or other inflammatory conditions. Given several failed antipsychotic medication trials before, and positive response to clozapine, Mr. L, his brother, and his psychiatrist agreed that the known benefits of clozapine to the patient outweighed the potential risks as long as close monitoring of blood parameters. Mr. L’s brother, who was his health care proxy, was involved in extensive discussions about the risks of clozapine, and Mr. L agreed to the trial.

**Fig. 1:**
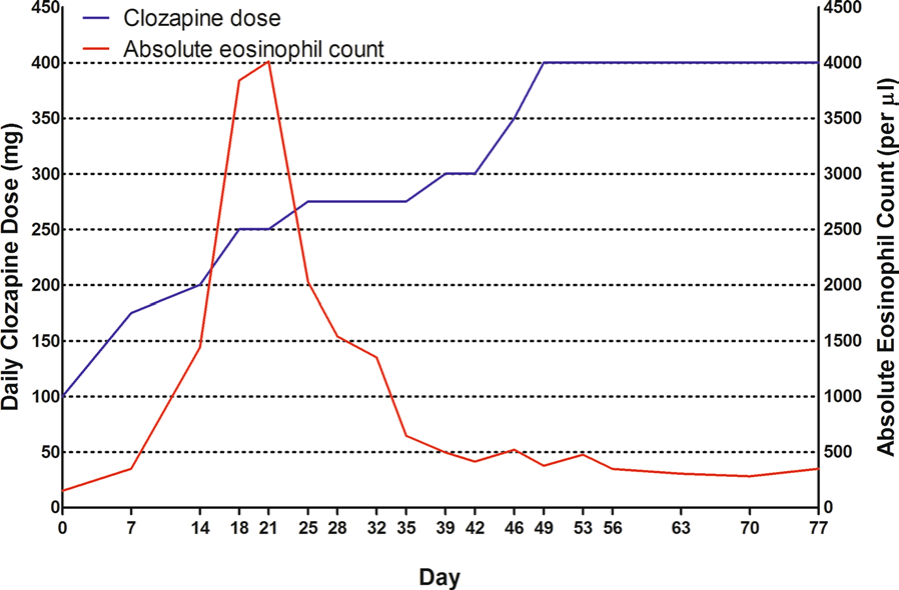
Changes in absolute eosinophil count after initiation of clozapine use

The recommendation of the hematology service was to continue clozapine treatment with close monitoring of complete blood count with differential test to track his eosinophil count even with the current level of eosinophilia since the patient was not showing signs of end-organ involvement. Figure 1 illustrates the course of Mr. L’s clozapine dosage and eosinophil counts throughout the clozapine trial and beyond. Over the next 3 weeks, clozapine was titrated slowly. Mr. L’s eosinophilia reached a peak of 4.0 × 10^9^/l, 34% of the total WBC count (11.8 × 10^9^/l), at a clozapine dosage of 250 mg/day, but decreased then and remained within normal limits 3 weeks later. The dosage was gradually raised as high as 400 mg/day. His psychotic symptoms got partial remission and continued to show no signs of end-organ inflammation at the time of discharge.

## Discussion and conclusions

The pathophysiology of clozapine-induced eosinophilia is still unknown, but resolution of eosinophilia despite ongoing clozapine treatment suggests the possibility of an acute allergic reaction [16]. Although the manufacturer of clozapine recommends discontinuation when the level of eosinophils is above 3 × 10^9^/l and eventually restarting when the level of eosinophils is below 1 × 10^9^/l, some experts suggest the potential consequences of discontinuation of clozapine outweigh this risk if all other medical conditions are excluded [17]. Signs or symptoms of organ inflammation are important for management of eosinophilia. In this case report, we demonstrated that if eosinophilia occurred without signs or symptoms of organ inflammation, it may be justified to continue clozapine use under careful monitoring.

## Abbreviation

ECT: electroconvulsive therapy
